# HIV Protease: Historical Perspective and Current Research

**DOI:** 10.3390/v13050839

**Published:** 2021-05-06

**Authors:** Irene T. Weber, Yuan-Fang Wang, Robert W. Harrison

**Affiliations:** 1 Department of Biology, Georgia State University, Atlanta, GA 30302, USA; ywang24@gsu.edu; 2 Department of Computer Science, Georgia State University, Atlanta, GA 30302, USA; rwh@gsu.edu

**Keywords:** HIV/AIDS, retroviral proteases, drug resistance, protease structures, antiretroviral inhibitors

## Abstract

The retroviral protease of human immunodeficiency virus (HIV) is an excellent target for antiviral inhibitors for treating HIV/AIDS. Despite the efficacy of therapy, current efforts to control the disease are undermined by the growing threat posed by drug resistance. This review covers the historical background of studies on the structure and function of HIV protease, the subsequent development of antiviral inhibitors, and recent studies on drug-resistant protease variants. We highlight the important contributions of Dr. Stephen Oroszlan to fundamental knowledge about the function of the HIV protease and other retroviral proteases. These studies, along with those of his colleagues, laid the foundations for the design of clinical inhibitors of HIV protease. The drug-resistant protease variants also provide an excellent model for investigating the molecular mechanisms and evolution of resistance.

## 1. Introduction

The HIV/AIDS pandemic was first recognized in the early 1980s as being due to infection by a novel retrovirus, termed human immunodeficiency virus type 1 (HIV-1). In the past four decades, about 33 million people have died from the disease. By current estimates, about 38 million people are infected with HIV [1]. Due to intense efforts by many experts in retrovirology, medicinal chemistry, enzymology, computational modeling, and structural biology, a number of antiretroviral drugs have been developed to target several different stages in the viral lifecycle, cell fusion and entry, and the activity of the three viral enzymes: protease (PR), reverse transcriptase (RT), and integrase (IN) [2]. These antiviral agents are highly effective in combination therapy. The current recommendations of the World Health Organization are described in [3]. In the absence of an effective vaccine for HIV, RT and IN inhibitors are used for pre-exposure prophylaxis. However, the long-term success of both antiviral therapy and prophylaxis is compromised by the prevalence of drug-resistant strains of the virus [4]. Rates of new HIV infections with transmitted drug resistance have increased in North America and Sub-Saharan Africa in recent years [5]. 

This review focuses on HIV-1 PR, which is a valuable target for antiretroviral drugs. The basic structure and function of this enzyme were determined in the late 1980s and early 1990s. PR is encoded in the viral genome and produced as part of the Gag-Pol precursor polyprotein. During the maturation stage of the viral lifecycle, PR is responsible for processing Gag and Gag-Pol precursors into mature viral proteins [6,7]. Due to its essential role in viral replication, HIV PR was quickly recognized as a potential target for the development of antiretroviral drugs [8,9]. PR was recognized as a member of the aspartic protease family due to the presence of the conserved catalytic residues Asp-Thr/Ser-Gly [10]. The mature PR is catalytically active as a dimer of two 99-residue subunits, and each subunit contains one copy of the catalytic triplet. PR recognizes specific amino acid sequences at the different cleavage sites in the Gag and Gag-Pol polyproteins and hydrolyzes the peptide bond to release the individual structural proteins and enzymes. The cleavage sites must be hydrolyzed in the correct sequential order to produce infectious virus [11,12,13]. From 1995 to 2006, nine antiviral inhibitors of PR were approved for HIV/AIDS therapy. Their long-term effectiveness for therapy is limited by undesirable side effects, inaccessible reservoirs of the virus, and the emergence of drug resistance. These problems have been addressed in recent studies of drug-resistant variants of PR and structure-guided designs of novel inhibitors for resistant virus. 

## 2. Historical Background: Structure and Specificity of HIV Protease

**Figure 1:**
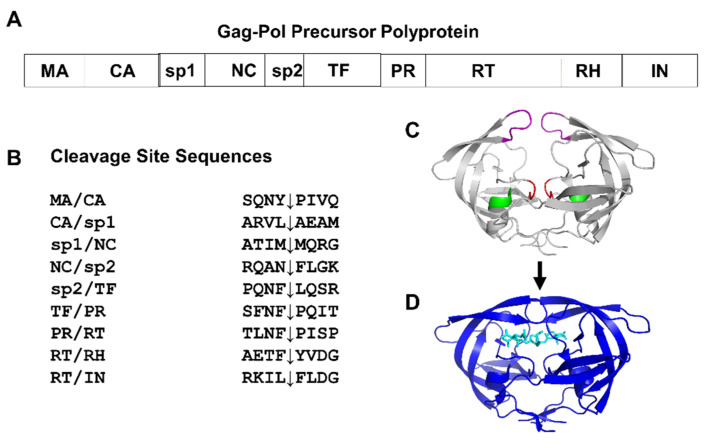
(**A**) The HIV-1 Gag-Pol polyprotein precursor is processed by PR during maturation to release individual structural proteins MA, CA, and NC, and enzymes PR, RT, and IN. (**B**) PR hydrolyzes the peptide bond, indicated by an arrow in the listed cleavage site sequences of Gag-Pol. (**C**) The dimer of mature PR (grey ribbons) exists in an open conformation in the absence of substrate or inhibitor. The conserved catalytic triplet of residues Asp-Thr-Gly is shown in red, the conserved triplet of Gly-Arg-Asn in the alpha helix is in green, and the Gly-rich ends of the flexible flaps are in purple. (**D**) The PR dimer (blue ribbons) bound to the peptide analog of the sp1/NC cleavage site (cyan sticks) has closed conformation flaps.

During the late 1980s and 1990s, studies of the structure and substrate specificity of HIV PR provided an important foundation for the development of antiviral protease inhibitors for the treatment of HIV/AIDS. Basic information on the structure and function of HIV PR is summarized in Figure 1. Dr. Steven Oroszlan and his colleagues in retrovirology pioneered many of these early studies [8]. Dr. Oroszlan’s group reported the genetic location and sequence of HIV-1 PR and its cleavage sites (Figure 1A,B) [14,15], the chemical synthesis of the PR gene for expression in *E. coli* [16], purification of the expressed PR [17], and a spectroscopic assay for its proteolytic activity [18]. He also collaborated in initial efforts to develop selective inhibitors of HIV-1 protease [19,20,21]. Moreover, he inspired several of the junior researchers in his group to pursue related research after they moved to other institutions. 

The crystal structure of HIV-1 PR was determined in 1989 by three different groups [22,23,24]. Later in the same year, the first crystal structure was reported for PR in complex with a substrate analog inhibitor [25]. In subsequent years, numerous structures became available for HIV PR bound to various inhibitors [26]. The PR dimer exists in a dynamic equilibrium between two distinct conformations as shown in Figure 1C,D [27]. When substrates or inhibitors bind, PR forms a closed conformation where the ligand lies in a cavity and interacts with the catalytic residues and the two flexible flaps. In the absence of substrate or inhibitor, the flaps move away from the catalytic site and assume an open conformation. The conformational dynamics of the flaps are important for the recognition of cleavage sites in the natural polyprotein substrates and their ordered cleavage [13]. Structural studies of HIV PR have identified key amino acids in the substrate-binding site and their interactions with substrate analogs. These structures were critical for the design of antiretroviral inhibitors. 

These early investigations into the sequence, structure, and substrate specificity of HIV-1 PR and how it compares with other retroviral proteases gave fundamental insights into the relationships among different PRs and their substrates. Overall, the amino acid sequences of different retroviral PRs share about 20–30% identity [28]. Conserved regions include the catalytic triplet (Asp-Thr/Ser-Gly), the C-terminal triplet at the start of the alpha helix (Gly-Arg-Asn/Asp), and the glycine-rich flaps.

Dr. Oroszlan and others analyzed the specificity of HIV-1 PR for various peptide substrates and compared PRs from HIV-1 and -2 [29,30,31,32,33]. The amino acid sequences of HIV-1 and -2 PRs share about 40% identity. The two PRs show similar, although not identical, specificities for peptide substrates. In particular, some clinical inhibitors, such as amprenavir, which were designed to target HIV-1 PR, are less effective on HIV-2 PR [34]. HIV PR and related retroviral PRs preferentially cleave the peptide bond between hydrophobic amino acids at P1 and P1’ in the standard nomenclature for protease substrates [35], including the unusual hydrolysis of the peptide bond between the aromatic side chains of Phe or Tyr at P1 and Pro at P1’. 

In parallel, other studies compared HIV-1 PR to the PRs of various mammalian retroviruses. The studied PRs were from equine infectious anemia virus [36,37], murine leukemia virus [38,39,40,41], bovine leukemia virus [42], and mouse mammary tumor virus [43]. The early findings are summarized in [44]. Later investigations from Dr. Oroszlan and his collaborators addressed the structure and substrate specificity of PR from a different human retrovirus, human T-cell leukemia virus [45,46]. A separate series of studies focused on mutational analysis of the Rous sarcoma virus (RSV) PR in relation HIV-1 PR [47,48,49,50,51,52]. This analysis extended to drug-resistant mutations of HIV-1 PR and their relation to substrate specificity [53,54]. Similar studies have continued in recent years [55,56]. Insights from these specificity studies informed the design of improved antiviral agents and also correctly predicted which residues might mutate into drug resistance. 

**Figure 2:**
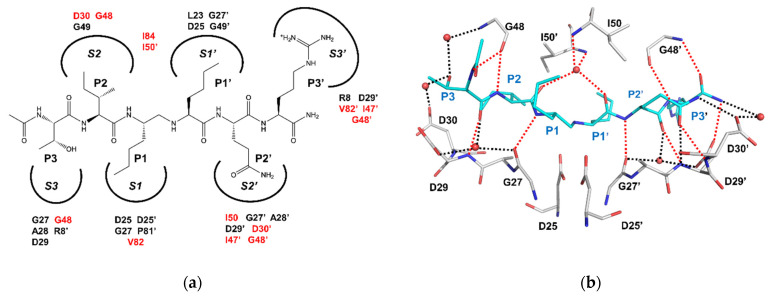
(**a**) Substrate peptide in the binding cavity of HIV-1 PR. P3 to P3’ amino acids are shown for a peptide analog of the sp1/NC cleavage site, T-I-Nle-Nle-Q-R, where Nle is norleucine, an analog of methionine, and non-hydrolyzable CH_2_-NH replaces the peptide bond between P1 and P1’. Each side chain of the peptide binds in pockets or subsites S3–S3’ (curved lines) in the PR dimer. PR residues contributing to the subsites are indicated. Residues that vary in different retroviral PRs are shown in red; (**b**) hydrogen bond interactions between PR (grey bonds) and the sp1/NC substrate analog (cyan bonds) are shown in an orientation approximately perpendicular to (**a**). Water molecules in the binding site are shown as red spheres. Hydrogen bond interactions are indicated as dotted lines. Red dotted lines show conserved interactions between main chain C=O and NH groups of PR and main chain groups of substrate analog. Black dotted lines indicate non-conserved hydrogen bonds.

The crystal structures reveal how HIV-1 PR binds the peptide analogs of substrate cleavage sites as illustrated in Figure 2. The dimer of HIV PR binds about six residues of peptide analogs of its substrate, where a non-hydrolysable group replaces the peptide bond between P1 and P1’. Each side chain of the peptide (P3–P3’) binds in a pocket or subsite (S3–S3’) formed by PR residues. The residues of the subsites comprise both conserved amino acids among related PRs and amino acids that vary in different PRs (Figure 2a). The variable residues in the substrate binding site are also mutated in drug-resistant HIV as described later. Mutations of these non-conserved residues are associated with major drug resistance in the clinic [57]. The structures of different PRs show a conserved series of hydrogen bond interactions between the main chain amide and carbonyl oxygen atoms of PR and the main chain atoms of substrate analogs (Figure 2b) [27]. The clinical inhibitors of HIV PR were designed to retain many of these hydrogen bonds, as described in the next section. 

## 3. Antiviral Protease Inhibitors for HIV/AIDS

**Figure 3:**
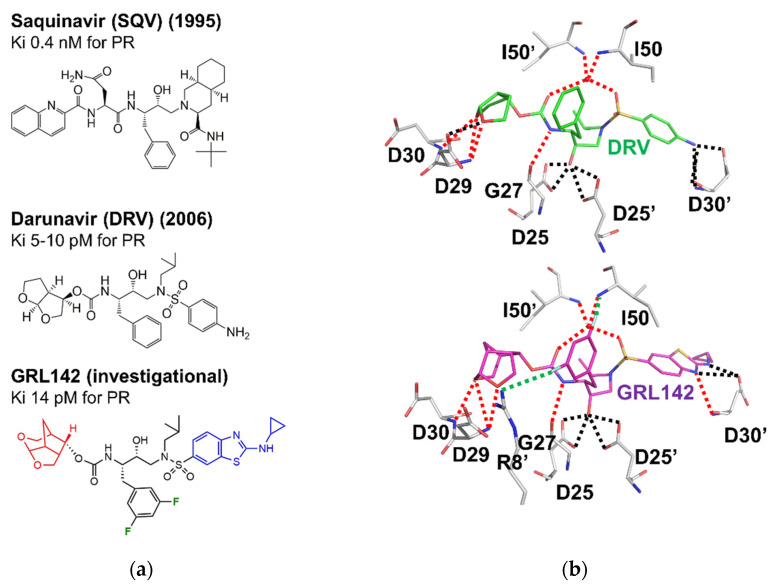
(**a**) Chemical structures of clinical inhibitor saquinavir (approved in 1995), clinical inhibitor darunavir (approved in 2006), and investigational inhibitor GRL142, colored to show differences from darunavir; (**b**) hydrogen bond interactions between PR (grey bonds) and inhibitors darunavir (top in green bonds) and GRL142 (bottom in magenta bonds). A key water molecule is shown as a red sphere. Hydrogen bonds are shown as dotted lines. Red dotted lines indicate interactions similar to those observed for peptide analogs (see Figure 2b). Green dotted lines indicate halide interactions. Black dotted lines indicate non-conserved hydrogen bonds.

The structures of HIV PR became the basis for ground-breaking efforts to develop antiviral drugs for HIV/AIDS [26]. The protease inhibitor, saquinavir, was first described in 1990 [58] and approved by the FDA for clinical use in 1995. This inhibitor and subsequent drugs were designed based on the structures of HIV PR with substrate analog inhibitors. Key constraints include the conserved set of hydrogen bond interactions observed between the main chain amides and the carbonyl oxygens of peptide analogs and the main chain groups in the PR binding site (Figure 2b). Currently, nine antiviral protease inhibitors are approved. All are peptidomimetics, except for tipranavir. The second generation of inhibitors was designed to target drug-resistant strains of the virus. The newest inhibitor, darunavir, was approved for clinical use in 2006 and shows the highest binding affinity of 5–10 pM for HIV protease. Darunavir, lopinavir, and atazanavir are currently recommended in second-line regimens for people failing first-line therapy with IN and RT inhibitors [3] and are available combined with RT inhibitors emtricitabine and tenofovir in a fixed dose regimen [59]. Selected antiretroviral PR inhibitors are shown in Figure 3. The design goal for darunavir was to incorporate chemical groups capable of mimicking the conserved hydrogen bonds in the structures of PRs with peptide inhibitors [60]. The rationale is that hydrogen bond interactions between the main chain atoms of PR and peptide analogs cannot easily be eliminated by mutations. This strategy has resulted in the development of several potent antiviral inhibitors derived from darunavir [61]. Recent designs, such as GRL142, incorporate fluorine to improve penetration of the central nervous system [62,63]. Inhibitors that can attack viral reservoirs in the brain have promise for the treatment of neurocognitive disorders associated with HIV/AIDS [64]. 

## 4. HIV Drug Resistance

**Figure 4:**
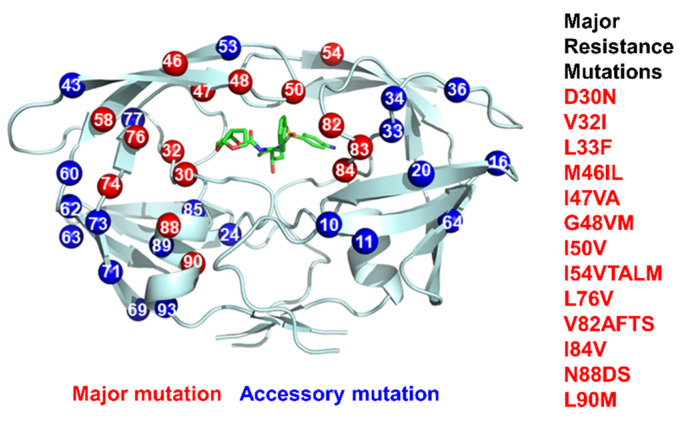
Drug-resistant mutations (DRMs) mapped on the structure of the HIV PR dimer (grey ribbons) in complex with darunavir (green sticks). Major DRMs are numbered red spheres, and minor or accessory mutations are blue spheres. Major DRMs are listed on the right.

HIV occurs in two types, HIV-1 and HIV-2. HIV-1 genomes comprise three main groups, M, N, and O, along with many subtypes and variants. This genomic diversity exacerbates the problems for treatment and accelerates drug resistance [65]. Drug-resistant strains of HIV evolve rapidly due to the high rate of replication, error-prone RT, and viral recombination [66,67]. Genotype analysis of newly infected patients and those failing antiviral regimens is an important component of clinical treatment [4]. Mutations associated with drug resistance are compiled in [57] and the Stanford HIVdb [5,68]. Figure 4 illustrates the drug-resistant mutations (DRMs) and their location in the PR structure. Individual mutations that are strongly associated with resistance to one or more clinical inhibitors are designated as major DRMs. High level resistance, however, generally requires an accumulation of multiple mutations, including additional ‘minor’ or accessory mutations, as well as the major DRMs. 

Resistance to PR inhibitors arises primarily by mutations in PR, although other mutations also occur in its Gag and Gag-Pol substrates [69]. Major mutations associated with resistance are often deleterious for viral replication [70]; however, viral fitness can be restored by additional, compensatory mutations [71,72]. The molecular mechanisms observed for PRs bearing single major mutations were reviewed in [73]. Major DRMs can directly influence the binding of inhibitors by altering amino acids in the inhibitor-binding site of PR, or they can have indirect effects by altering residues at the subunit–subunit interface in the dimer or altering the conformational dynamics of PR. The role of distal mutations is often obscure. In practice, mutations accumulate in the viral genome, and antiviral therapy drives the evolution of mutants with increasingly higher levels of resistance that thrive in the presence of antiviral drugs. 

The genotype and phenotype data available in HIVdb [5,68] have proved valuable for computational analysis of resistance. We have used machine learning with a unified encoding of sequence and structure to predict resistance and to select mutants representing high levels of resistance for detailed biochemical and biophysical studies [74,75,76]. Mutants PRS17 and PRS5B were chosen by this procedure and confirmed to show poor binding of clinical inhibitors [77,78,79]. Our recent graph theoretical analysis of genotype data mapped PR mutants onto different branches of a minimum spanning tree, based on their distances from the combined structure–sequence metric. The minimum spanning tree was hypothesized to be a proxy for the evolution of drug resistance [80]. Mapping drug resistance along the branches of the tree showed that the evolution of drug resistance first occurs as a ‘just resistant’ mutation followed by further evolution toward being highly resistant. Shah et al. [80] hypothesized that there is a selective pressure for higher levels of resistance to minimize the probability of a revertant mutation. We exploited these genotype–phenotype data to generate and evaluate hypotheses about drug resistance and PR variants. 

Highly resistant mutants observed in patients failing therapy exhibit affinity for inhibitors several orders of magnitude worse. Selected examples are given in Table 1 with their mutations and inhibition values for darunavir. These mutants contain 17-22 amino acid substitutions relative to a reference sequence for subtype B. Clinical mutant PR20 was initially reported in 2007 to show poor inhibition by darunavir [81]. PRdrv4 was identified in a pediatric patient and is characterized by its structure and affinity for darunavir [82]. Mutants PRS17 and PRS5B were selected by computational analysis of genotype-resistance data as described above and represent examples with high-level resistance to 6 and 5 clinical inhibitors, respectively. 

**Figure 5:**
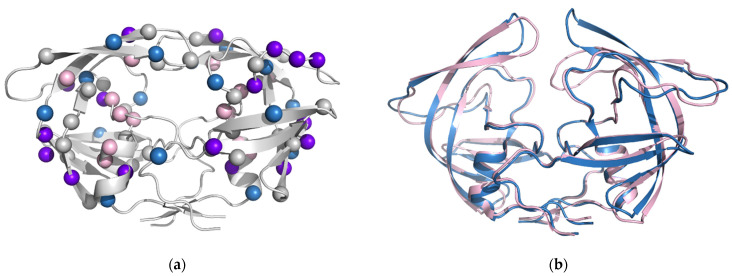
(**a**) Sites of DRMs (spheres) mapped on the PR dimer (grey ribbons). PR20 and PRS17 show different sets of mutations. Mutations only in PR20 are pink, mutations only in PRS17 are light blue, mutations common to PR20 and PRS17 are purple, and other DRMs are grey; (**b**) different flap conformations are observed for PR20 (pink ribbons) and PRS17 (light blue ribbons) dimers in the absence of inhibitors. PR20 has one flap in an extended open conformation and one flap protruding into the active site. PRS17 has a more symmetrical arrangement with two open flaps.

We investigated the structures and enzymatic properties of PR20, PRS17, and PRS5B in order to elucidate the molecular basis for their drug resistance [78,79,84]. These two highly resistant mutants show different distributions of mutations; only half of their mutations are in common (Figure 5a). PR20 includes mutations of four amino acids in the inhibitor-binding site. In particular, mutations I47V and I84V introduce smaller amino acids and create a larger binding cavity, which is proposed as a major contribution to the observed poor affinity for inhibitors. The other 17 mutations show coordinated effects that remodel the interior of the protein and indirectly influence inhibitor binding. In contrast, PRS17 has only two mutations in the inhibitor-binding cavity, G48V and V82S; however, distal mutations exert significant effects on the conformational dynamics. Moreover, PRS17 shows improved binding to substrate analogs compared to the wild-type enzyme, which is likely to contribute to drug resistance [85]. 

Differences in the conformational dynamics of the flaps are common in highly drug-resistant variants. NMR studies demonstrated that both PR20 and PRS17 exhibit differences in the flap dynamics relative to the wild-type PR. The flaps of drug-resistant mutants tend to occupy the open conformation in the absence of bound substrates or inhibitors, whereas the conformational equilibrium of wild-type enzyme tends toward the closed conformation even in the absence of ligands [13,78,84]. A greater variety of open conformations has been captured in crystal structures of highly resistant mutants compared to the wild-type PR, as illustrated in Figure 5b. PR20 exhibited an extremely open conformation of the flaps and also an unusual conformation with one flap tucked into the active site. PRS17 shows a distinctive curl at the tip of the flaps. Due to the highly dynamic nature of the flaps in resistant mutants, new inhibitors have been designed to introduce additional interactions with the flaps. Some inhibitors also incorporate fluorine, which improves penetration of the central nervous system. We are currently evaluating the effectiveness of the new antiviral inhibitors for PR20 and other highly resistant mutants [86,87]. One example, GRL142, is shown in Figure 3. This inhibitor exhibits 20-fold better affinity than darunavir for extremely resistant mutant PR20 [87] and is promising for further clinical development. 

## 5. Conclusions

Our current research into the mechanisms of drug resistance and the development of improved antiviral inhibitors for HIV PR is firmly based on many of the original findings of Steven Oroszlan and his colleagues. Early studies of the substrate specificity of HIV PR combined with knowledge of the crystal structure of PR with peptide analogs were vital to the design of potent antiretroviral inhibitors. Moreover, the differences seen in the amino acid sequences of different retroviral PRs bear strong similarities with mutations in drug-resistant HIV PR. This similarity demonstrates the importance of comparative studies of related proteins to understanding the evolution of resistance. 

## Figures and Tables

**Table 1:** Highly resistant mutants of HIV-1 protease.

Protease	*K*d DRV (nM)	Relative Kd	Amino Acid Substitutions Major Resistance Mutations
Wild-Type	0.005	1.0	
^a^ PR20	41	8200	L10F, I13V, I15V, D30N, V32I, L33F, E35D, M36I, S37N, I47V, I54L, Q58E, I62V, L63P, A71V, I84V, N88D, L89T, L90M
^b^ PRdrv4	35	7000	L10F, I13V, K14R, V32I, L33F, K45T, M46I, I47V, I54L, I62V, L63P, A71T, I72T, G73T, V77I, P79S, I84V, L90M
^c^ PRS17	50	10,000	L10I, K20R, E35D, M36I, S37D, M46L, G48V, I54V, D60E, I62V, L63P, A71V, I72V, V77I, V82S, L90M, I93L
^d^ PRS5B	4.0	800	L10I, V11I, E21D, A22V, L24M, E35N, M36I, S37D, R41K, M46L, I54V, Q61H, I62V, I63P, I64V, I66V, A71V, I72T, G73T, N83D, I84V

Data are taken from the following references: ^a^ [83], ^b^ [82], ^c^ [77], ^d^ [79].
